# Post combustion CO_2_ capture with calcium and lithium hydroxide

**DOI:** 10.1038/s41598-022-14235-5

**Published:** 2022-06-22

**Authors:** Maria Antonietta Costagliola, Maria Vittoria Prati, Giuseppe Perretta

**Affiliations:** Istituto di Scienze e Tecnologie per L’Energia e la Mobilità Sostenibili—National Research Council of Italy, viale Marconi, 4, 80125 Naples, Italy

**Keywords:** Environmental impact, Carbon capture and storage

## Abstract

A small-scale plant was built for measuring the ability of solid sorbents towards the capture of carbon dioxide (CO_2_) in exhaust flue gas from an internal combustion engine. The investigated sorbents were calcium and lithium hydroxides. Both sorbents are low cost and used in the breathing gas purification systems. The carbonation capacity of each sorbent was measured for different sorbent granulometry (pellets and powder), different temperature (from ambient up to 300 °C), gas space velocity, moisture content and chemical composition of the gaseous stream. The aim was, in fact, to expose the sorbents to a gas stream with chemical and physical parameters close to those at the exhaust of an internal combustion engine. Carbonation capacity was measured with a double technique: on-line by continuously CO_2_ measurement with a non-dispersive infrared analyzer and off-line by using scanning electron microscopy on carbonated sorbents. Experimental results showed good CO_2_ uptake capacity of calcium hydroxide at low temperature (between 20 and 150 °C). Performance improvements came from the fine granulometry due to the increased exposed surface area; moreover, the presence of the moisture in gas stream also enhanced CO_2_ capture. The presence of sulphur dioxide and nitric oxide, instead, greatly decreased the carbonation capacity of sorbents.

## Introduction

Reducing greenhouse gas (GHG) emissions has become a necessity to avoid the climate change and its catastrophic impacts. Human activities are adding in the atmosphere enormous amounts of greenhouse gases thus greatly influencing the earth's temperature. Transportation of people and goods plays a key role for their contribution to carbon dioxide (CO_2_) emissions in the atmosphere.

The European Environment Agency has estimated that, in 2019, transport sector (including road transport, domestic navigation and aviation, railways) emits almost 824 Mt CO_2_, covering almost 28% of global CO_2_ emissions in Europe. Moreover, GHG emissions from international shipping and aviation represent 4.7% and 4.5% of global CO_2_, respectively^1^.

The European Commission adopted a series of legislative proposals to achieve climate neutrality in the EU by 2050, including the intermediate target of an at least 55% net reduction in greenhouse gas emissions by 2030. Among these proposals, reducing greenhouse gas emissions from transport (e.g. through CO_2_ emission standards for vehicles) is crucial. This objective, in contrast with the constantly growing demand for national and international transportation, implies the necessity to apply a lot of technological measures, each able to reduce CO_2_ emissions from means of transport. CO_2_ reductions can be mainly reached by implementing energy efficiency measures in order to decrease fuel use. Among the most promising measures, there are the hybrid electric propulsion, the optimization of vehicles operation modes such as the speed, the load and the voyage planning^2,3^. Moreover, the replacement of fossil fuels with renewable fuels (such as biofuels) and with alternative energy (wind and solar power, fuel cell) should mitigate the environmental impact of transport^4^.

Another possible approach to mitigate CO_2_ emissions in the atmosphere is the post combustion capture, i.e. CO_2_ is captured from the exhaust flue gas of an internal combustion engine. In a recent study^5^, it was demonstrated that post-combustion carbon capture is a valid transition solution to lower the CO_2_ emissions in the short term; they showed that a system using the amine solvent for chemical absorption can capture up to 90% of the whole emissions. However, the CO_2_ capture with amine solution presents a great disadvantage: it is highly energy intensive, because of the high regeneration energy requirement. For this reason, in the last years a growing interest was toward adsorption process which uses novel solid sorbents characterized by a greater capacity and selectivity for CO_2_ capture and also by ease of handling and reduced costs^6^.

To this aim, two solid absorbents, calcium and lithium hydroxides, are investigated for CO_2_ capture in flue gas. Calcium Hydroxide (Ca(OH)_2_) has many environmental applications. It is, in fact, used for flue gas treatment to reduce the emission of acidic gases (HCl, SOx, and NOx) ant it is also an effective solvent to absorb CO_2_. As CO_2_ absorber, it was studied mainly in aqueous solution. Previous study demonstrated the good efficiency of Ca(OH)_2_ aqueous solution when exposed at high CO_2_ gas concentration (30%v)^7^. As solid sorbents, the main applications available in literature are referred to high temperatures (almost 800 °C for absorption and 1000 °C for desorption)^8,9^. The absorption rates are relatively fast and dropped off due to an impermeable build up of carbonates on the surface of solid sorbents. Because of the heterogeneous nature of the reactions, in fact, the formation of a surface product layer of carbonates around the reacting particles at the beginning of a reaction is unavoidable^10^.

In the past, main applications of lithium hydroxide (LiOH) for CO_2_ uptake were in space life support systems, space shuttle environmental control and submarine scrubbing systems. The irreversible and exothermic reaction between LiOH and CO_2_ occurs at room temperature with a high absorption capacity because of the low molar mass of LiOH. The adsorption capacity of LiOH is strongly dependent from moisture content in the CO_2_ stream^11^. A study on LiOH adsorbed zeolytes demonstrated that at ambient temperature the maximum CO_2_ uptake occurred at 65–70% relative humidity values^12^. Formation of lithium hydroxide monohydrate as an intermediate compound has been postulated to explain the effect of water vapor on the reaction^13^. For this reason, it can be easily exploited for CO_2_ removal from moisture-rich exhaust gas of an internal combustion engine.

The two solid sorbents were examined in a laboratory pilot plant which simulates a CO_2_ absorber unit for a flue gas of an internal combustion engine. Sorbents are arranged in a fixed bed reactor and several operation parameters (space velocity, granulometry, temperature, moisture content) were investigated.

## Materials and methods

### Solid sorbents

CO_2_ capture was studied with two low cost solid sorbents: soda lime and lithium hydroxide. Among the current uses of both hydroxides, there is the breathing gas purification systems for medical devices, spacecraft, submarines and rebreathers to remove carbon dioxide from exhaled gas. Soda lime (Medisorb by GE Healthcare) is composed by 75%w Ca(OH)_2_, 3%w NaOH and water. It appeared as white pellets (mesh 2.5–5 mm) classified as irritant for eyes, skin and respiratory system (Table 1). It has a relative density of 2 g/cm^3^ and is slightly soluble in water. Pellets were also grinded to obtain a fine size of soda lime (powder, mesh 10–50 μm) to be exposed to CO_2_ uptake.

**Table 1:** Chemical and physical characteristics of sorbents.

	Soda lime (Ca(OH)_2_)	LiOH anidrous	LiOH monohydrate (LiOH∙H_2_O)
Purity, %	> 75	> 98	≥ 98
Granulometry	pellets	pellets	Powder (~ 30–250 μm)
Molecular weight, g/mol	74,093	23,95	41,96
Density, g/cm^3^	2	2,54	1,51
Boiling point	–	–	100 °C @ 1013 hPa
Hazards identification	Skin Corrosive I B, H314 Causes severe skin burns and eye damage	Acute toxicity, Oral (Category 4), H302 Skin corrosion (Category 1B), H314	Acute toxicity, Oral (Category 4), H302 Skin corrosion (Category 1B), H314

Two formulations of lithium hydroxide were tested: anidrous (LiOH) and monohydrate (LiOH∙H_2_O). LiOH was provided in pellets and LiOH∙H_2_O in powder, mesh 30–250 μm (Sigma-Aldrich, reagent grade ≥ 98%, Table 1). Their classification indicates acute toxicity and skin corrosion. Relative density of LiOH is 2.54 g/cm^3^, and that of LiOH∙H_2_O is 1.51 g/cm^3^. LiOH anidrous and monohydrate were tested separately and mixed (50–50%v). The mixed solid is characterized by a mixed granulometry (powder and pellets) which has two main advantages. The first is the greater surface area compared with anidrous pellets, which obviously enhances the CO_2_ uptake; the second is a lower water content compared to hydrate solid, which avoids the undesired effect of grain agglomeration.

**Figure 1:**
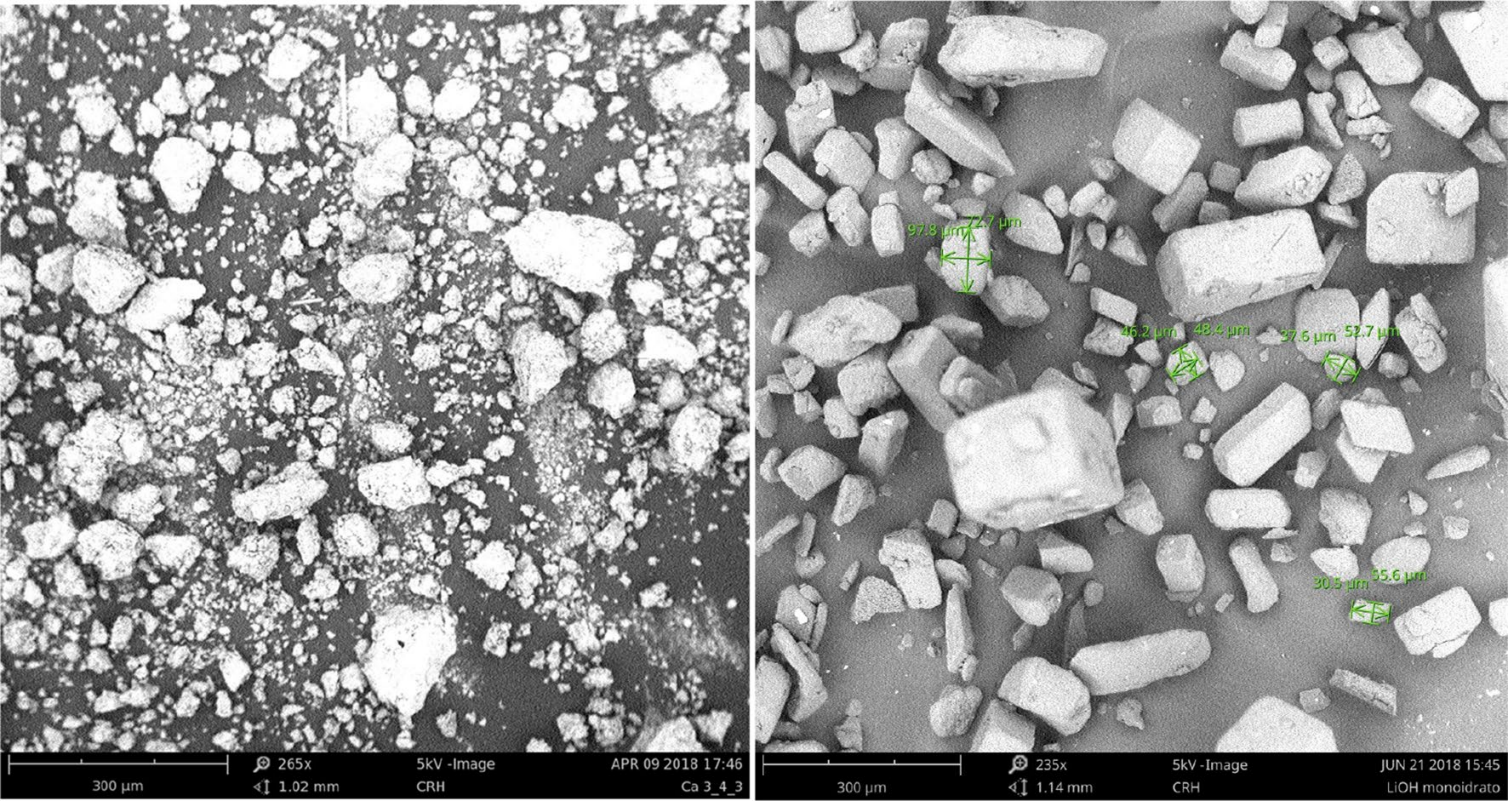
SEM image of soda lime in powder (left side) and LiOH·H_2_O (right side).

Figure 1 reports SEM image of both sorbents.

#### Pilot-plant description

**Figure 2:**
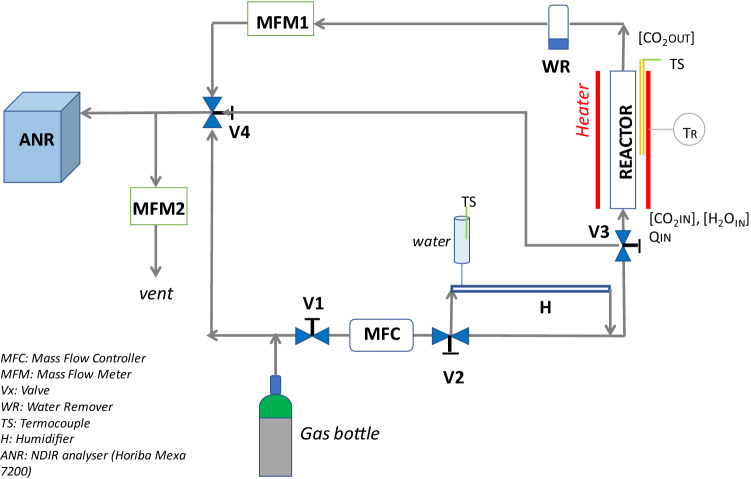
Pilot plant scheme.

Figure 2 schematizes the laboratory plant developed for the experimental activity. A quartz cylindrical reactor (length = 50 cm, diameter = 1.4 cm) was used for testing the solid sorbents. The sorbents were inserted inside the reactor and fixed by using fiberglass tips.

Although gas flow was set at 8 l/min, space velocity (volumetric flow per volume unit, a key parameter for the after-treatment catalyst design) was changed by varying the height of sorbent inside the reactor.

The reactor was housed in a cylindrical heater able to set the temperature from the ambient up to 1000 °C. A thermocouple is inserted into the furnace to control the temperature reached near the reactor. The inlet flow to the reactor was regulated by a Mass Flow Controller (0–20 l/ min, Bronkhorst), connected to the gas bottle containing CO_2_; the output flow of the reactor was sent to a moisture separator, a flow meter and then to the Non-Dispersive Infra-Red (NDIR) analyzer by Horiba. On the sampling line of the analyzer, a vent was inserted for the overflow exiting the reactor. The valves V3 and V4 were inserted to allow the measurement of the gas inlet concentration. In this case, in fact, the gas bottle is directly connected with the analyzer, without going through the reactor.

In order to analyze the effect of water content, the inlet gas, before to reach the reactor, was diverted by a three-way valve (V2) to the humidifier (Permapure's NafionTM MH-Series Humidifier tube). In this way, the sorbent was studied in dry and wet conditions. The distilled water for the humidifier is contained in a graduated syringe and a thermocouple records its temperature. At the humidifier outlet, the wet stream to be treated was sent to the reactor.

The water content entering the reactor is measured by the Horiba analyzer using NDIR (Non-Dispersive InfraRed Detector) detectors. Furthermore, the total volume of water absorbed by the system during the whole duration of the test is measured by the difference between the initial and final volume contained in the graduated syringe.

#### Carbonation capacity measurements

Capacity of carbonation was continuously measured throughout the measurement of CO_2_ concentrations upstream and downstream the reactor according the following Eq. (1).

1 $$C=\frac{Q {[{CO}_{2}]}_{in}d}{m MW}{\int }_{0}^{t}\left(1-\frac{{\left[{CO}_{2}\right]}_{out}}{{\left[{CO}_{2}\right]}_{in}}\right)dt$$

where: C: Carbonation capacity, moleCO_2_/kg sorbent, Q: volumetric flow rate, m^3^/min, [CO2]_in_: inlet CO_2_ concentration, ppmv, [CO2]_out_: outlet CO_2_ concentration, ppmv, d: CO_2_ density, 1976 g/m^3^, m: mass of sorbent, kg, MW: CO_2_ molecular weight.

Carbonation capacity increases with time, up to reach the maximum value where CO_2_ concentration at the exit of reactor is equal to the inlet one. In the experimental tests, maximum carbonation capacity was considered when [CO_2_]_out_ is 95% of [CO_2_]_in_.

Carbon uptake of sorbents was also evaluated off line throughout scanning electron microscope (SEM Phenom Pro X) analysis of exposed hydroxides. The SEM is equipped with an Energy Dispersive Spectrometry (EDS) detector for elemental analysis. Low acceleration voltage of 5 kV was used for imaging in order to prevent back scattering phenomena^14^. Voltage of 15 kV was, instead, used for EDS analysis. For SEM–EDS analysis, not exposed samples were compacted into tablets whereas exposed samples were fixed on the aluminum holder throughout a carbon sticker.

## Results and discussion

### Calcium and Lithium hydroxide performance

**Figure 3:**
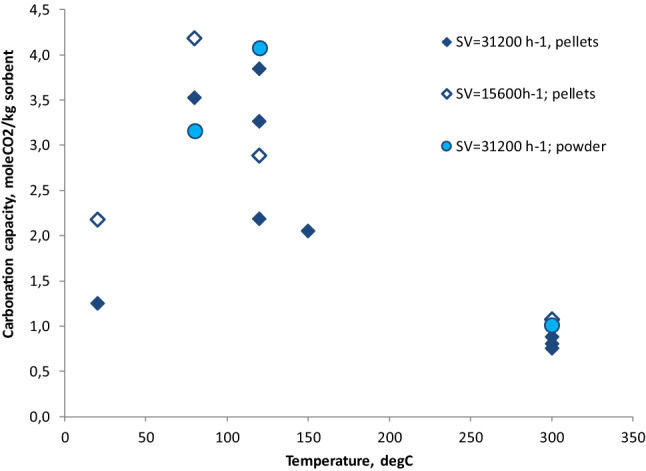
Soda lime carbonation capacity as a function of the temperature.

CO_2_ uptake of Ca(OH)_2_ was tested in the range between ambient temperature and 300 °C. Results are reported in Fig. 3 which shows carbonation capacity (molCO_2_/kg sorbent) in dry conditions for both pellets and powder. Ca(OH)_2_ pellets were tested for two space velocity (SV): 31,200 and 15,600 h^−1^. Looking at all data, the highest carbonation efficiency is observed in the range of temperatures from 80 to 100 °C. For temperature higher than 100 °C, the carbonation capacity decreases because of the loss of sorbent humidity. As mentioned above, in fact, soda lime contains almost 10–15% of water which promotes the carbonation reaction. Indeed, where the temperature makes possible the water evaporation (> 100 °C), the carbonation reaction is not enhanced.

At the investigated temperatures, main carbonation reaction involves directly Ca(OH)_2_ according following Eq. (2).

2 $${\text{Ca}}\left( {{\text{OH}}} \right)_{{2}} + {\text{ CO}}_{{2}} = {\text{ CaCO}}_{{3}} + {\text{ H}}_{{2}} {\text{O}}$$

It takes place until 350–400 °C; at higher temperatures, instead, dehydration of Ca(OH)_2_ to CaO can occur^15^. Some papers indicate that the optimal temperature of Ca(OH)_2_ carbonation is almost at 200 °C^16^. Carbonation capacity trend as a function of the temperature, reported in Fig. 3, confirms this statement.

**Figure 4:**
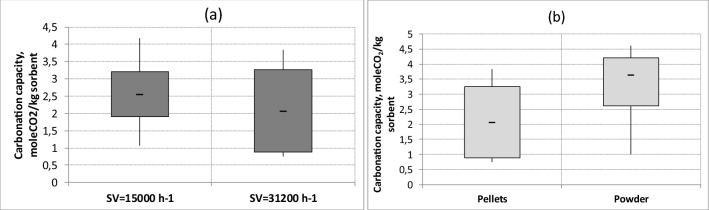
Influence of space velocity (**a**) and granulometry (**b**) on soda lime carbonation capacity.

The carbonation capacity of calcium hydroxide is dependent on Space Velocity. The variation of this parameter was obtained by varying the height of the reactor filling (10 and 20 cm for 15,600 and 31,200 h^−1^, respectively). By grouping the data with the same space velocity and same granulometry (pellets), it should be stated that an increase in space velocity determines an average reduction in carbonation capacity of almost 25% (Fig. 4a). Therefore, a longer contact time favors the CO_2_ capture capacity.

For stating the influence of sorbent granulometry, data at space velocity of 31,200 h^−1^ were grouped according sorbent granulometry, pellets and dust (Fig. 4b). Fine granulometry involves an increment of carbonation capacity of almost 40% compared to pellets. Main reason is the increased surface area of dust which is involved in the carbonation reactions. It has to be noted that the fine granulometry corresponds to the best absolute result obtained with soda lime (almost 3.6 molCO_2_/kg sorbent corresponding to 35% of maximum possible carbon uptake by the tested sorbent mass).

**Figure 5:**
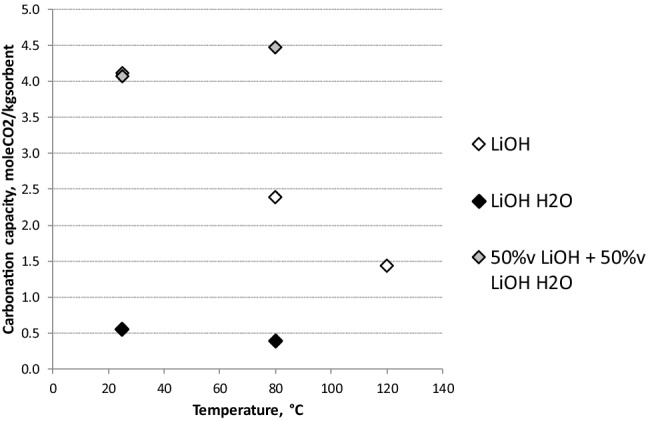
Carbonation capacity of LiOH anhydrous and monohydrate as a function of the temperature.

Figure 5 shows carbonation capacity measured with LiOH anydrous and monohydrate, as a function of the temperature. Data are referred to a space velocity of 32,100 h^−1^. When increasing temperature, carbonation capacity of LiOH decreases: the highest capacity of 4 molCO_2_/kg sorbent is measured at the ambient temperature. At T = 120 °C, the carbonation capacity drops to 1.5 mol CO_2_/kg sorbent. The increasing of temperature, in fact, does not promote the formation of monohydrate hydroxide and then carbonation rate decreases ^17^.

Main reactions (Eqs. 2 and 3) occurring for CO_2_ absorption involve the production of the intermediate hydroxide monohydrate; then, it reacts with CO_2_ to form lithium carbonate.

2 $${\text{2LiOH}}\left( {\text{s}} \right) \, + {\text{ 2H}}_{{2}} {\text{O}}\left( {\text{g}} \right) \, = {\text{ 2LiOH}}\cdot{\text{H}}_{{2}} {\text{O}}\left( {\text{s}} \right)$$

3 $${\text{2LiOH}}\cdot{\text{H}}_{{2}} {\text{O}}\left( {\text{s}} \right) \, + {\text{ CO}}_{{2}} \left( {\text{g}} \right) \, = {\text{ LiCO}}_{{3}} \left( {\text{s}} \right) \, + {\text{ H}}_{{2}} {\text{O}}\left( {\text{g}} \right)$$

Therefore, the efficiency of CO_2_ absorption is strictly influenced by water adsorption rate on the LiOH surface. As consequence, the percentage carbonation capacity measured for anydrous LiOH is almost 13% of maximum CO_2_ mass which is possible to capture, by considering the stoichiometry. Moreover, it was observed that, despite the presence of water, the carbonation capacity of LiOH monohydrate is always lower than 0.5 mol CO_2_/kg sorbent. This is caused by the too fine granulometry of the available sorbent; in fact, since CO_2_ absorption reactions lead to the water formation in addiction to that already present in the LiOH⋅H_2_O, the granules agglomerate forming macro-granules characterized by a reduced surface area. Better performance than the two pure sorbents were obtained by mixing the two hydroxides (50% v/50% v). In this way the new mixed sorbent was characterized by a mixed granulometry (pellets and powder) and also by a reduced water content compared with the pure lithium monohydrate hydroxide. The carbonation capacity of mixed sorbent is greater than 4 mol CO_2_ / kg sorbent, reaching the best performance with lithium sorbent (26% of CO_2_ captured compared with the maximum allowed).

#### SEM/EDS analysis

**Figure 6:**
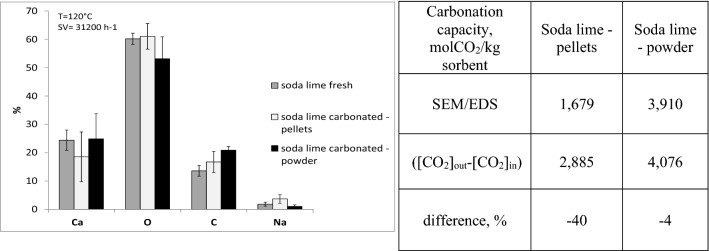
Elemental analysis of fresh and carbonated soda lime by SEM/EDS.

Some samples of soda lime exposed to CO_2_ gas stream were also analyzed with SEM to obtain elemental composition. Figure 6 shows the elemental composition of soda lime exposed to a gaseous mixture containing CO_2_ at 20%v in nitrogen, at the reactor temperature of 120 °C and with a Space Velocity of 31,200 h^−1^. The graph shows the compositions for the fresh (not exposed) sample and for 2 carbonated samples: original particle size (pellets) and fine particle size (powder). The data correspond to 20 scans average for each sample analyzed. SEM analysis confirms that the fine grain size shows a greater carbonation capacity. In fact, the average percentage concentration of C measured in the exposed soda lime powders is 21%; the soda lime of original grain size, instead, has a C content of approximately 16,7%.

Considering that the fresh sample has an initial carbon content of 13,6%, the C trapped is 7,3% for the powders and 3,1% for the pellets. Starting from these percentages, the carbonation capacities were estimated in the two samples of soda lime, considering that the number of moles of CO_2_ trapped by the sorbent coincides with the number of carbon atoms. The results are compared with carbonation capacity estimated by online CO_2_ measurement downstream of the reactor (left side of Fig. 6). The greater exposure of the fine particle size to the gaseous stream allows to obtain a good agreement between the direct measurement of the carbonation capacity and the indirect measurement by means of elemental SEM/EDS analysis (difference of 4%). When analyzing the pellets, the difference is greater (+ 40% of the direct measurement compared to the SEM).

### Influence of the moisture on the carbonation capacity of Ca(OH)_2_

Solid sorbents capable of capturing CO_2_ in gaseous effluents show the best performance in the presence of moisture. Water has a catalytic effect in the process of CO_2_ absorption by hydroxides^12^. The steam, in fact, performs a catalytic action for the diffusion of CO_2_ through the upper carbonated layer of the sorbent^17–19^. Furthermore, it has been shown that in high temperature applications, the steam can induce the widening of the average size of the absorbent pores, enhancing the capture of CO_2_^20,21^.

It was demonstrated that the reaction mechanism is dependent by alkalinity of adsorbed water on Ca(OH)_2_ surface ^22^. Equations from (4) to (9) describe the possible reactions in presence of water.

4 $${\text{Ca}}\left( {{\text{OH}}} \right)_{{{2}({\text{s}})}} = {\text{ Ca}}^{ + + }_{{({\text{aq}})}} + {\text{ 2OH}}^{ - }_{{({\text{aq}})}}$$

5 $${\text{CO}}_{{{2}({\text{aq}})}} + {\text{ H}}_{{2}} {\text{O}}_{{({\text{aq}})}} = {\text{ H}}_{{2}} {\text{CO}}_{{{3}({\text{aq}})}}$$

6 $${\text{H}}_{{2}} {\text{CO}}_{{{3}({\text{aq}})}} = {\text{ H}}^{ + }_{{({\text{aq}})}} + {\text{ HCO}}_{{3(aq)}}^{ - }$$

7 $${\text{H}}_{{2}} {\text{CO}}_{{{3}({\text{aq}})}} = {\text{ H}}^{ + }_{{({\text{aq}})}} + {\text{ CO}}_{{3(aq)}}^{2 - }$$

8 $${\text{CO}}_{{{2}({\text{aq}})}} + {\text{ OH}}^{ - }_{{({\text{aq}})}} = {\text{ HCO}}_{{3(aq)}}^{ - }$$

9 $${\text{Ca}}^{2 + }_{{({\text{aq}})}} + {\text{ CO}}_{{3(aq)}}^{2 - }= {\text{ CaCO}}_{{{3}({\text{s}})}}$$

For higher water alkalinity, carbonate ion is predominant and carbonation reaction (9) is favored; otherwise, bicarbonate ion is predominant (reactions (6) and (8)) and dissolved in water layer. Reactions of adsorption and hydration of CO_2_ and the formation of carbonate ion are very fast, whereas the dissolution of Ca(OH)_2_ may be slow, depending on the adsorbed humidity.

In order to study the effect of moisture on CO_2_ capture, wet conditions were realized in small-scale reactor by using a NafionTM tube humidifier, positioned upstream of the reactor inlet. In particular, it was possible to achieve 2 and 5% as humidity (H). It has to be noted that the addition of moisture in gas stream adds also the advantage to simulate chemical gas composition closer to those of an internal combustion engine exhaust (water concentration between 5–10% v).

Each condition was examined with the Ca(OH)_2_ sorbent both in pellet and in powder, in the temperature range between ambient temperature and 150 °C. The Space Velocity used for these tests was 31,200 h^−1^.

**Figure 7:**
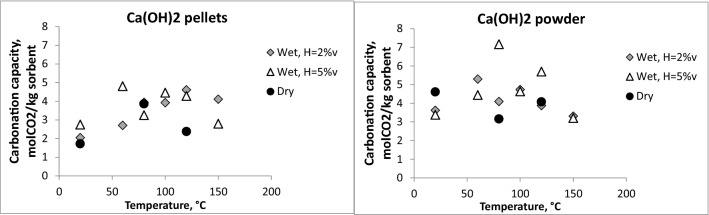
Carbonation capacity of soda lime in wet and dry conditions as a function of the temperature.

Figure 7 reports data of wet and dry carbonation capacity as a function of temperature. By increasing the water content, an improvement of carbonation capacity is clearly visible only for pellets. Soda lime powder shows a higher CO_2_ uptake only in correspondence of few temperatures (80 and 120 °C).

**Figure 8:**
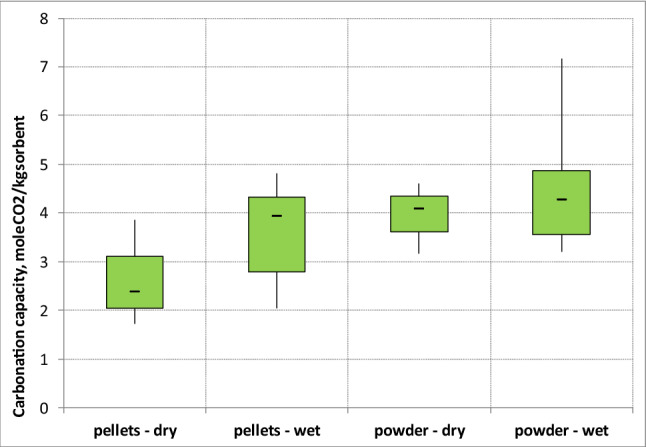
Average carbonation capacity of soda lime in wet and dry conditions.

In order to deeply investigate the influence of humidity, the box plot of Fig. 8 summarizes the average carbonation capacity measured in dry and wet conditions (H = 2% v and 5% v). The data were grouped according to the granulometry of the sorbent. It is evident that in the investigated experimental conditions, the humidification of the gas stream introduced appreciable benefits for the pellets. In this case, in fact, the average carbonation capacity goes from 2,3 molCO_2_/ kg sorbent to 3.9 molCO_2_/ kg sorbent. For the powder size, on the other hand, the improvement in the carbonation capacity for the humidification of the gaseous current is low due to agglomeration phenomena of the sorbent with a consequent decrease in the surface area.

These results are comparable with literature. Recently^23^, investigated the CO_2_ capture by using a fluidized bed of calcium hydroxide, previously humidified. They analyze the capacity of CO_2_ uptake at ambient temperature, atmospheric pressure and 1%v CO_2_ inlet concentration. When increasing the relative humidity from 24 to 100%, carbonation capacity doubled up to almost 0,3 molCO_2_/kg sorbent. A similar research carried out by^24^, showed that if Ca-based sorbent is 8 h pre-hydrated, carbonation capacity is almost 6 molCO_2_/kg sorbent corresponding to almost 10 times higher than dry value.

### Influence of chemical composition of gas stream on CO_2_ capture

The interference of other gas on CO_2_ capture was studied. The attention was focused on some compounds normally present in the exhaust flue gas of internal combustion engine. To this aim, carbonation capacity of soda lime was monitored by using the following gas mixtures:

Mix 1: CO_2_ (10-20%v) in nitrogen

Mix 2: CO_2_ (10%v), CO (0,5%v), C_3_H_8_ (330 ppmv), NO (1000 ppmv) in nitrogen

Mix 3: CO_2_ (6%v), SO_2_ (400 ppmv) in nitrogen

Mix 2 has a chemical composition close to that of the engine exhaust, whereas mix 3 was used to analyze the interference of sulphur on the carbonation capacity of soda lime.

**Figure 9:**
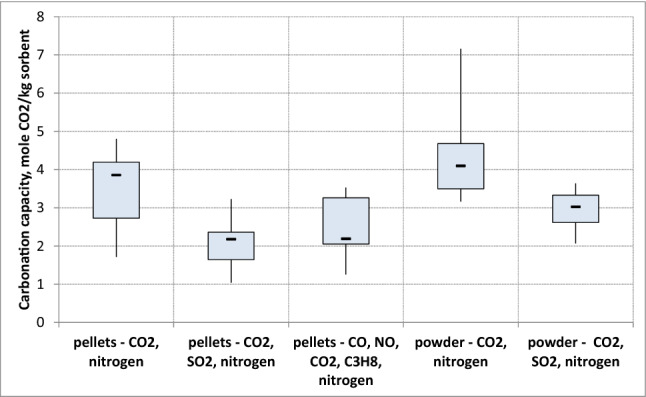
Ca(OH)_2_ carbonation capacity with gas mixtures of different chemical composition.

Figure 9 reports average carbonation capacity of Ca(OH)_2_ as a function of chemical composition of gas mixture. Data are grouped according sorbent granulometry (pellet or powder). It should be noted a lower CO_2_ uptake in presence of other gaseous compounds compared with the binary mixture of CO_2_ and nitrogen. Compounds such as NO, CO, C_3_H_8_ and SO_2_ reduce carbonation capacity more than 60%.

**Figure 10:**
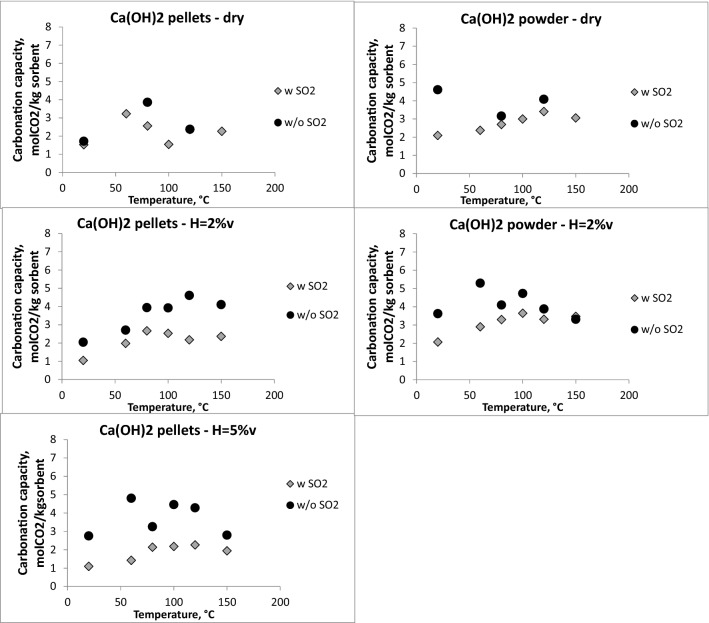
Carbonation capacity of Ca(OH)_2_ w and w/o SO_2_.

SO_2_ interference was deeply investigated varying the reactor temperature and moisture content in gas stream (Fig. 10). Each graph reports carbonation capacity with and without SO_2_ as a function of the temperature. Moisture content and granulometry are fixed.

The efficiency reduction in CO_2_ capture increases as the humidity of the gas stream increases. In dry conditions, differences in the carbonation capacity due to the presence of SO_2_ is almost 15% for pellets and 29% for powder. The greatest difference in carbonation capacity is, however, measured at a water vapor concentration of 5%v. In this case reduction of carbonation capacity drops of almost 50% compared with data measured in absence of SO_2_ (data available only for Ca(OH)_2_ in pellets). Many literature studies have shown that the carbonation efficiency with calcium-based sorbents is significantly reduced by the presence of SO_2_, due to the irreversible reaction between Ca and SO_2_ to form CaSO_4_^25,26^. Furthermore, the sulphate forms a surface layer on the sorbent particles and prevents the diffusion of CO_2_ through the pores of the sorbent itself.

## Conclusions

CO_2_ capture from solid sorbents seems to be an interesting technique to control CO_2_ emissions from internal combustion engine. The paper investigated the performance of low cost, not toxic and safe sorbents.

Main results show that soda lime (mainly calcium hydroxide) was found to be a good sorbent to be used for capturing CO_2_ from effluents at low temperatures (between 20 and 150 °C).

The parameters that significantly influence the carbonation capacity are:

- grain size: fine grain size has a greater surface area available for carbonation reactions;
- Humidity: increasing the humidity of the current to be treated increases the carbonation capacity;
- Presence of other compounds: the presence of other compounds such as SO_2_ and NO significantly reduces the CO_2_ capture efficiency

The use of fine grain size sorbents presents the disadvantage of agglomeration phenomena of the sorbent itself, especially in humid conditions.

The negative effect found on the carbonation capacity from the presence of SO_2_, as this can compete with CO_2_ in the absorption, leads us to say that a CO_2_ capture system of this type must be installed downstream of a removal system of sulfur oxides (and preferably also nitrogen oxides), such as a scrubber or an SCR system.

Carbonation capacities of the order of 5 mol of CO_2_ per kg of sorbent have been obtained. The use of an inert porous substrate could significantly improve the performance of hydroxides in terms of CO2 capture efficiency, since it could avoid blockages due to sorbent agglomeration phenomena. Even if it involves an increase in the weight of the overall sorbent.

## Data Availability

The datasets generated and/or analysed during the current study are not publicly available due to the rules of the funding research contract but are available from the corresponding author on reasonable request.
